# Improper disposal of face masks during COVID-19: unheeded public health threat

**DOI:** 10.11604/pamj.2021.38.366.29063

**Published:** 2021-04-14

**Authors:** Fisseha Shiferie

**Affiliations:** 1 Department of Epidemiology and Biostatistics, Addis Continental Institute of Public Health, Addis Ababa, Ethiopia

**Keywords:** COVID-19, face mask, improper disposal, waste management

## Abstract

Ethiopia confirmed its first case of the novel coronavirus (COVID-19) pandemic in March 2020. As a means to tackle the spread of the virus, the government opened a campaign for the public to exercise hygiene measures such as washing hands frequently and physical distancing. A few weeks later, a five-month State of Emergency (SOE) was declared, and several businesses, schools and theatre halls were closed. People were advised to work from home, including permitting telecommuting for many government officials. However, mainly due to fear of economic crisis, the government was forced to release the lockdown and mass wearing of face masks has become a requirement. As health professionals and the government insist that people make use of facemasks, it is also equally important to give guidance on how to dispose of them safely because face masks that are disposed improperly have the potential in spreading SARS-CoV-2. The urgency of this comes in people's violations of rules when it comes to disposing of masks they used. Providing awareness creation programs about the negative impact of contaminated face masks on the health of individuals and introducing laws that can prohibit improper disposal of used personal protective equipment (PPEs) are among the solutions discussed in this manuscript that could help reduce the problem.

## Commentary

Ethiopia confirmed its first case of the novel coronavirus (COVID-19) pandemic in March 2020. As a means to tackle the spread of the virus, the government opened a campaign for the public to exercise hygiene measures such as washing hands frequently and physical distancing. A few weeks later, a five-month State of Emergency (SOE) was declared, and several businesses, schools and theatre halls were closed. People were advised to work from home, including permitting telecommuting for many government officials. However, mainly due to fear of economic crisis, the government was forced to release the lockdown that was imposed in Addis Ababa and considered other options. Together with the aforementioned prevention mechanisms, mass wearing of face masks has become a requirement [1].

**Utilization of face masks in public areas and its unique importance for dense cities:** currently, the “wear a face mask in public areas” order seems arguably successful in Addis Ababa. There are reasons for this. Because its implementation has been followed-up by the police and other law enforcement bodies, the fear of getting arrested or penalized might have been the primary reason. The ease of use and affordable price of masks is another motivator. At pharmacies, a disposable surgical facemask goes for just 10 Br. Another reason might have been the tremendous effort of healthcare professionals and public campaigns in creating awareness about the importance of wearing facemasks. The wide usage of this simple preventive measure is more important in dense cities like Addis Ababa as it is hard to adhere to the rule of physical distancing. This is especially the case in market places, bus and taxi stations and places of worship. The frequent water outage in the city also makes hand hygiene a rather difficult task. Wearing face masks will thus remain the most important preventive measure as businesses and schools reopen. Despite controversies in some countries about the importance of face masks, recent studies show that they play a pivotal role in the prevention and control of infectious respiratory disease transmission, including COVID-19. There is a general perception in Germany that the mandatory use of face masks in public reduces COVID-19 incidences considerably. After face masks became mandatory between 1^st^ April and 10^th^ April 2020, the number of new infections fell almost to zero. Eventually, face masks became mandatory in all federal states between 20^th^ April and 29^th^ April 2020 [2].

**Improper disposal of face masks is a threat to public health and the environment:** as health professionals and the government insist that people make use of facemasks, it is also equally important to give guidance on how to dispose of or recycle them safely. One of the areas where the environmental impacts of COVID-19 are most pronounced is in waste management. This is a major environmental concern because of the surge in the demand for and the use of plastic products, protective gears, personal protective equipment (PPE), disposable life support equipment and general plastic supplies like syringes, all used in the prevention and treatment of the virus [3]. The urgency of this comes in people´s violations of rules when it comes to disposing of masks they used. It has now become common to see used masks scattered everywhere - on sidewalks and in parking lots. Although rampant littering of plastic bottles, papers and banana skins was the norm before the pandemic, the magnitude of the problem is worsening with the throwing of potentially COVID-19 infected masks in public and open spaces.

Infectious waste is characterized as any material that is suspected to contain pathogens (bacteria, viruses, parasites or fungi) in sufficient concentration or quantity to cause disease in susceptible hosts. It also comprises waste contaminated with blood, bodily fluids, tissues, organs and sharp objects [4]. Public health experts say that improperly discarded masks could be potential sources of the virus if people come into contact with them. The poor and inadequate waste management strategies within developing and least developed countries contribute to a higher threat of community spread of COVID-19 [3]. For instance, in cities like Addis Ababa, where thousands of individuals (including children), make their lives working in the streets, coming into contact with these contaminated face masks becomes easy. In the worst cases, they might even be tempted to use them, if not to wear on their faces but to make use of them in some other way. An environmental disaster seems to be looming in this regard. There is a high chance for masks to get washed away and end up in oceans, rivers and lakes, affecting flora and fauna negatively. Improper disposal of biomedical waste is responsible for soil and groundwater pollution and adversely affects the biota [5]. World Wide Fund for Nature (WWF) Italy estimated that for Phase 2, in which production and social activities will be progressively restarted, 1 billion masks and half a billion gloves will be needed per month. If even 1% of the masks are not disposed properly, this would result in as many as 10 million face masks per month dispersed in the environment. Considering that the weight of each mask is about 4 grams, this would result in the dispersion of over 40,000 kilograms of plastic poses a dreadful future [6].

**How to dispose of face masks safely during the COVID-19 pandemic?** In this commentary, I have tried to suggest easy and implementable solutions for the safe disposal of face masks as they have the potential in spreading or encouraging the spread of the SARS-CoV-2. It does not require to be an environmental health expert or possess a special knowledge to keep our surrounding clean and appealing. All we need is to be responsible individuals in our routine daily activities. The least we can do is to keep the used mask in our cars, luggage or plastic bag until we get a rubbish bin where we can safely dispose them. If we do not find one on our way, we can throw them into a closed bin when we get back home. Providing awareness creation programs would certainly be one way of curbing the problem. The negative impact contaminated face masks would cause on the health of individuals, especially to vulnerable groups, and the harm they would bring to the environment can be concisely presented in short videos or posters. The program can reach to the public through available communication channels such as radio, television or social media platforms.

Preparing guidelines about safe disposal of facemasks in particular and personal protective equipment (PPE) in general (if not done yet) is highly needed now than ever. While guidelines regarding proper medical waste management are available in healthcare settings in Addis Ababa, guidelines for proper face mask disposal are either unavailable or have not reached communities well. Introducing laws that can prohibit improper disposal of used PPEs could also help reduce the problem. The police force and other law enforcement bodies need to closely monitor individuals´ practices regarding improper face mask disposal in every corner of the city. Those who go against the law should get appropriate punishment. Important here is the need for more rubbish bins. Awareness campaigns and laws will go unheeded if the necessary infrastructure is not in place to make it easy for people to carry out such tasks.

Elimination of COVID-19 can only be achieved with vaccines and there is a huge demand currently for available vaccines. It has been a long time now since the pandemic has significantly changed our way of life. The 'new normal' has officially begun in our city since March 2020. As it is compulsory to wear face masks in public spaces, it should also be our responsibility to dispose of them safely and properly. Unless we do this, we will continue to live under threat to our lives.
